# A path analysis model suggesting the association of information and beliefs with self-efficacy in osteoporosis prevention among middle-aged and older community residents in urban Shanghai, China

**DOI:** 10.1371/journal.pone.0211893

**Published:** 2019-02-07

**Authors:** Yingchao Cui, Zijun Xu, Yue Shi, Yingyan Wu, Cheng Lv, Qiuming Shen, Tian Shen, Yong Cai

**Affiliations:** 1 School of Medicine, Shanghai Jiao Tong University, Shanghai, China; 2 School of Public Health, Shanghai Jiao Tong University, Shanghai, China; Shanghai Diabetes Institute, CHINA

## Abstract

**Background:**

Osteoporosis is a chronic disease whose prevention is more effective than treatment, but it may be necessary to change people’s self-efficacy to prevent this condition. This article aimed to study the pathway among information, beliefs and self-efficacy in osteoporosis prevention, and support further intervention.

**Methods:**

A cross-sectional study was conducted among community residents over 40 years old from two volunteer communities in urban Shanghai, China. Of 450 middle-aged and older community residents who volunteered to participate in the study, 421 (93.5%) finished the field survey effectively.

**Results:**

62.9% of the residents were females. Their mean age was 64.4 ± 11.2 years. The residents showed low knowledge of osteoporosis-related information, and the mean percentage of correct response was just 61.2%. In univariate analysis, information (univariate β = 0.27, 95% CI = 0.15–0.38) and beliefs (univariate β = 0.31, 95% CI = 0.25–0.38) were associated with self-efficacy. Multivariate analysis showed that information (multiple β = 0.19, 95% CI = 0.09–0.36) and belief (multiple β = 0.30, 95% CI = 0.23–0.36) remained significant. And in the path analysis, self-efficacy was significantly predicted by beliefs (β = 0.81, p<0.001).

**Conclusions:**

The study highlighted the urgency of conducting the osteoporosis preventive health promotion among middle-aged and older people as their lack of information and low level of beliefs and self-efficacy about osteoporosis prevention. Future interventions should focus on improving beliefs, especially perceived benefits, perceived threats, and action clues, on osteoporosis prevention in this group.

## Introduction

Osteoporosis is a common and easily overlooked metabolic bone disease [1]. In 1991, a Consensus Development Conference defined osteoporosis as a “systemic skeletal disease characterized by low bone mass and microarchitectural deterioration of bone tissue, with a consequent increase in bone fragility and susceptibility to fracture risk” [2]. This definition was subsequently accepted by the World Health Organization (WHO) in 1994 [3] and was widely used until a revision in 2000 which defined osteoporosis as a “skeletal disorder characterized by compromised bone strength predisposing to an increased risk of fracture” [4]. The prevalence among people over 40 years old in mainland China was 24.62% [5]. Fracture is a common complication of osteoporosis, and brings heavy burden to family and society [6–7].

Osteoporosis is therefore an important health issue among middle-aged and older people. Currently, many studies have shown that lack of physical activities and low consumption of calcium are significantly associated with osteoporosis and its complications [8–10]. In addition, measures such as using combination of drugs, calcium and vitamin D or even having surgery [11–12] can repair fractures properly, but cannot completely cure osteoporosis. There have been many studies on osteoporosis prevention, but their target population was usually osteoporosis patients or high-risk groups. The latest meta-analysis and systematic review [13] has showed that the prevalence rates of osteoporosis has increased with age, the latest prevalence of osteoporosis in people who are middle-aged and older community residents is estimated to be more than twice the prevalence in 2006 (34.65% vs. 15.7%) [14]. Therefore, the prevalence of osteoporosis among the Chinese elderly population was very high and it is necessary to reduce the risk of osteoporosis in whole population, especially among people over 40 years old [15]. At that point, osteoporosis prevention in community is beneficial and worthy of attention.

In the community, residents who have been diagnosed with osteoporosis will pay more attention to the risks of osteoporosis and change their behavior with doctor's help. However, those who have not been diagnosed may ignore the possible health risks of not changing their behavior to prevent osteoporosis effectively. Changing and measuring human behavior is a very complex issue, and thus we decided to use Albert Bandura’s social learning theory [16] to study the possibility of self-efficacy on behavior choice, especially the association of information and beliefs with self-efficacy.

According to Bandura’s theory, self-efficacy is individuals’ subjective judgment of their ability to perform a specific behavior, or their self-confidence in their ability to perform a particular behavior and achieve the desired result. People tend to avoid tasks and situations which they consider to be beyond their ability, but undertake action within their capabilities. Bandura [17] argued that perceived performance expectations determined the extent to which difficulties can be overcome. People with stronger perceived efficacy expectations will work harder to change their behavior, so self-efficacy may be the positive predictor of behavior [18].

Research suggested it necessary to expand the study of osteoporosis knowledge, especially to improve its effect on disease prevention and early diagnosis, which plays an important role in preventing osteoporosis [19]. A study showed there were significantly positive correlations among osteoporosis-related knowledge, health belief, and self-efficacy in middle-aged women [20]. Study also demonstrated that expectation factors of health beliefs, including relative benefit, self-efficacy, and health motivation, had a mediation effect between knowledge and prevention behaviors in osteoporosis prevention [21]. In summary, most studies stay on the surface to discuss their relationship, and do not go deep into analyzing the intricate pathways between information, beliefs and self-efficacy.

This study described osteoporosis related information, beliefs and self-efficacy among middle-aged and older community residents in Shanghai, China. We hypothesized that information and beliefs are correlated, while information and beliefs directly affect self-efficacy. On the basis of this, the article aimed to deeply study the pathways among them by performing structural equation modeling (SEM).

## Methods

### 2.1 Study site and ethics

The study was conducted in urban Shanghai, China, in 2016. Shanghai is the largest and the most developed city in China with a serious aging population[22]. Shanghai was selected because the city government has focused on osteoporosis prevention in recent years and there have been several interventions [23]. Understanding residents’ self-efficacy can identify potential problems and support future interventions in the area.

The study was reviewed and approved by the Ethics Committee of the School of Public Health, Shanghai Jiao Tong University. Before enrolment in the study, all participants were given information about the study objectives and procedures, and potential risks and benefits of participation, and they provided written informed consent at the beginning of the questionnaire.

### 2.2 Study population and sampling size

Convenience sampling method was conducted in the study. Residents over 40 years were enrolled from two volunteered communities in urban Shanghai from July to September 2016.

The prevalence of osteoporosis ranged from 24.5% to 44.2% [24–25] among all reports in Shanghai. Assuming the prevalence of osteoporosis of 35% among residents over 40 years old, an α of 0.05, and a relative sampling error of 0.15P, we calculated a required sample size of approximately 400 to allow for the larger sampling error of the convenience samples procedure and a non-response rate of 20%. Of the 450 older community residents who volunteered to participate in the study, 436 (96.9%) were eligible for participation and of those, 421 (93.5%) finished the field survey effectively. Each participant was paid the equivalent of 30 Chinese Yuan (1 USD = 6.89 CNY) as compensation for travel expenses.

### 2.3 Data collection

We used a one-to-one interview that the surveyor asked the question, and then wrote down the answer given by the residents over 40 years old. The content of the questionnaire was selected from the Osteoporosis Knowledge Test (OKT) [26], the Osteoporosis Health Belief Scale(OHBS) [27] and the Osteoporosis Self-Efficacy Scale(OSES) [28], with the permission of the authors, together with written informed consent and some demographic information. The demographic information included age, gender, height, weight, education, marriage status, working conditions (full/part time, unemployed), monthly income and the constructs of information, beliefs and self-efficacy. The three scales were tested in preliminary research and showed good fitness based on reliability and validity analysis [26–28]. The three scales were translated into Chinese and also showed good fitness among the middle-aged and elderly people in community [29–31]. We changed part of the multiple-choice questions from OKT into true or false questions, modified some items to fit with Chinese lifestyles and rearranged the sequences to ensure that there were not too many consecutive questions with the same answer. We changed the OSES answer sheet to a Likert-type scale with answers from 1 to 5 for ‘not at all confident’ to ‘very confident’.

Trained surveyors went to community health service centers to conduct one-to-one questionnaire interviews in the presence of the community teams. Prior to participation, we explained the survey aims and general content to each subject and emphasized that participation was voluntary and anonymous. Each questionnaire took approximately 15 minutes to complete.

### 2.4 Measures

#### 2.4.1 Information

Osteoporosis-related information was measured using the Osteoporosis Knowledge Test(OKT)[26] including 20 items, among which seven had possible responses of “yes”, “no” or “do not know” and the other 13 were multiple-choice questions with three possible answers plus “do not know”. Correct answers were given a score of one, while wrong or “do not know” answers scored zero. First indicator containing seven items was related to etiological information (e.g., “Do you think menopausal women are more likely to get osteoporosis?”). Individual question scores were summed and converted into a total score for etiological information (Cronbach’s alpha coefficient = 0.608; range 0–6). The second indicator included five items and was related to clinical-relative information (e.g., “Osteoporosis can be diagnosed by?”). Individual item scores were summed to produce a total score to represent clinical-relative information (Cronbach’s alpha coefficient = 0.573; range 0–5). The other indicator containing eight items was related to prevention information (e.g., “Which of the following is the recommended amount of calcium intake for an adult?”). Individual question scores were summed and converted into a total score for prevention information (Cronbach’s alpha coefficient = 0.508; range 0–8). Higher scores indicate students have access to more information.

#### 2.4.2 Beliefs

Beliefs in preventative actions against osteoporosis was measured by the Osteoporosis Health Belief Scale(OHBS)[27], which was adapted to use a five-point Likert-type scale (1 = strongly disagree, 2 = disagree, 3 = neutral, 4 = agree, 5 = strongly agree). The first index was perceived threats of osteoporosis, which contains two items (e.g. “Do you think your chances of getting osteoporosis are high?”). The sum of the scores of these two items served as the index of perceived threats (Cronbach’s alpha coefficient = 0.486; range 2–10). The second index was perceived benefits and contained three items (e.g., “Do you think regular exercise prevents problems that would happen from osteoporosis?”). The sum of the score of these three items served as perceived benefits (Cronbach’s alpha coefficient = 0.586; range 3–15). The third index was of perceived barriers and included three items (e.g., “Do you think calcium rich foods cost too much?”). The sum of the scores of the three items was taken as perceived barriers (Cronbach’s alpha coefficient = 0.641; range 3–15). The fourth index was action clues, including four items (e.g., “Do you look for new information related to health?”). The sum of the scores of the four items was taken as action clues (Cronbach’s alpha coefficient = 0.782; range 4–20). Higher scores indicate more efficient osteoporosis preventive beliefs.

#### 2.4.3 Self-efficacy

Self-efficacy in performing osteoporosis preventive behaviors was measured by the Osteoporosis Self-Efficacy Scale(OSES)[28], which was adapted to use a five-point Likert-type scale (1 = not at all confident, 2 = not confident, 3 = neutral, 4 = confident, 5 = very confident), and contained six items. The first three items represented exercise self-efficacy (e.g., “If it were recommended that you exercise at least three times this week, how confident or certain would you be that you could?”). The sum of the scores of the three items indicated exercise self-efficacy (Cronbach’s alpha coefficient = 0.928; range 3–15). The other three items represented Ca-intake self-efficacy (e.g., “If it were recommended that you take calcium supplements if you don’t get enough calcium from your diet this week, how confident or certain would you be that you could?”). The sum of the scores of the three items indicated Ca-intake self-efficacy (Cronbach’s alpha coefficient = 0.704; range 3–15). A higher score indicated more self-efficacy to apply osteoporosis preventive behaviors.

### 2.5 Statistical analysis

The database was established using Epidata3.0. Data were analyzed with the Statistical Program for Social Sciences version 20.0 (SPSS version 20.0) for Windows. Measurement data were listed by mean and standard deviation (SD) based on the distribution of data. Enumeration data were listed by frequency and relative frequency. Chi-square test and univariate linear regression were used for statistical inference. Structural equation modeling (SEM) was used to examine the hypothetical pathway model using Amose 20.0. SEM compares a proposed hypothetical model that can elucidate a relationship with a set of actual data. Pathway model fit was assessed using the comparative fit index (CFI), the root mean square error of approximation (RMSEA), and the maximum likelihood chi-square values/degrees of freedom ratio [32]. The CFI compares the proportional improvement in the model relative with a null model, and values greater than 0.9 indicate a good fit. The RMSEA value accounts for model complexity. Value lower than 0.05 indicates a good fit and value of about 0.08 or less would indicate a reasonable error of approximation [33]. A non-significant likelihood ratio chi-square test suggests the good model fit, but chi-square is sensitive to sample size, therefore, a χ^2^/df ratio of 3 or less indicates acceptable fit [32–33]. A preliminary confirmatory factor analysis (CFA) path model was constructed to examine predictors of osteoporosis self-efficacy among middle-aged and older community residents.

## Results

### 3.1 Social-demographic characteristics of the participants

A total of 421 community residents completed the survey, with an average age of 64.4 years (SD = 11.2; range: 41–95), and the majority (62.9%) were female. More than 80% of participants were married (82.2%) and fewer than 20% had a degree (15.4%), defined as a junior college or college graduate or above. Most participants (80.5%) were not at work and 64.1%earned an average personal monthly income of 3,100–6,000 Chinese Yuan (1 USD = 6.89 CNY). The self-efficacy score among females (Mean = 22.87, SD = 4.36) was significantly higher than that among males (Mean = 21.58, SD = 4.90). At the same time, the participants who were not at work showed better self-efficacy compared with participants who were at work. (p<0.01) (Table 1)

**Table 1:** Demographic characteristics and their associations with self-efficacy of the participants (N = 421).

Characteristics variables	Number of the participants	Self-efficacy	P-value
	N(row%)	Mean ± SD	
**Age groups(years)**			0.21
40–65	242(57.5)	22.64±4.41	
>65	179(42.5)	22.07±4.83	
**Gender**			0.005**
Male	156(37.1)	21.58±4.90	
Female	265(62.9)	22.87±4.36	
**BMI**			0.848
<18.5	15(3.6)	22.13±5.72	
18.5–24.9	268(63.7)	22.32±4.52	
≥25	138(32.8)	22.57±4.65	
**Education**			0.396
Low(junior high school and below)	194(46.1)	22.21±4.57	
Medium(high school/special secondary school/ vocational school)	162(38.5)	22.77±4.58	
High(junior college/college graduate and above)	65(15.4)	22.02±0.60	
**Current Marriage status**			0.570
Married	346(82.2)	22.12±4.44	
Single	75(17.8)	22.45±4.64	
**Employment status**			0.015*
At work(full/ part time)	82(19.5)	21.29±4.37	
Not at work(retired/ laid-off)	339(80.5)	22.66±4.62	
**Monthly income(CNY)**			0.999
<3000	132(31.4)	22.39±4.15	
3001–6000	270(64.1)	22.40±4.82	
>6000	19(4.5)	22.42±4.65	

* p<0.05

**p<0.01

### 3.2 Information, beliefs associated with self-efficacy among participants

In univariate linear regression, both information and beliefs showed a statistical significance, and the univariate β were 0.27(95%CI = 0.15–0.38), 0.31(95%CI = 0.25–0.38) respectively. After adjusting for sex and working status, the multiple linear regression showed that both information and beliefs remained significant, and the multiple β were 0.19(95%CI = 0.09–0.36), 0.30(95%CI = 0.23–0.36) respectively. (Table 2)

**Table 2:** Information, beliefs associated with self-efficacy among participants (N = 421).

Variables	Univariate β(95%CI)	Adjusted β(95%CI)	Multiple β(95%CI)
Information	0.27(0.15–0.38)**	0.25(0.13–0.36)**	0.19(0.09–0.36)**
Beliefs	0.31(0.25–0.38)**	0.31(0.25–0.38)**	0.30(0.23–0.36)**

Note: 95% CI: 95% confidence interval. Adjusted β, β adjusted for sex, work status

Multiple β: B obtained from forward stepwise multivariate linear regression using significant variables of the univariate analysis as input

* p<0.05

** p<0.01.

### 3.3 Confirmatory factor analysis

The residents showed low knowledge of osteoporosis-related information: the average information test score was 12.24 correct responses out of a possible 20, with the mean percentage of correct response was just 61.2%(12.24/20). And the mean percentage of correct response to etiological information, clinical-relative information and prevention information were 68.7%(4.81/7), 52%(2.60/5) and 60.5%(4.84/8) respectively.

A preliminary confirmatory analysis was conducted to estimate the factor structure and relationships of the latent variables among the 421 participants. The means, standard deviations, ranges and factors loadings were listed in Table 3. All factor loadings except perceived barrier and information in the model were significant (p<0.05).

**Table 3:** Summary statistics and factor loadings of the HBM based on confirmatory factor analysis(N = 421).

Scales	Mean(95%CI)	SD	FL
Information (Range:0–20)	12.24(11.87–12.61)	3.87	0.115
Etiological information(Range:0–7)	4.81(4.64–4.97)	1.77	0.64**
Clinical-relative information(Range:0–5)	2.60(2.46–2.73)	1.42	0.66**
Prevention information (Range:0–8)	4.84(4.68–4.99)	1.63	0.75**
Beliefs (Range:12–60)	42.08(41.46–42.69)	6.40	0.814**
Perceived threats (Range:2–10)	6.24(6.03–6.44)	2.13	0.35**
Perceived benefits (Range:3–15)	11.30(11.08–11.52)	2.32	0.65**
Perceived barriers (Range:3–15)	8.81(8.55–9.07)	2.71	-0.08
Action clues (Range:4–20)	15.73(15.44–16.02)	3.05	0.82**
Self-efficacy (Range:6–30)	22.39(21.95–22.84)	4.60	
Exercise self-efficacy (Range:3–15)	11.04(10.74–11.34)	3.11	0.53**
Ca-intake self-efficacy (Range:3–15)	11.36(11.13–11.58)	2.32	0.80**

Note: SD, standard deviation; FL, factor loading

**, p<0.01.

**Fig 1:**
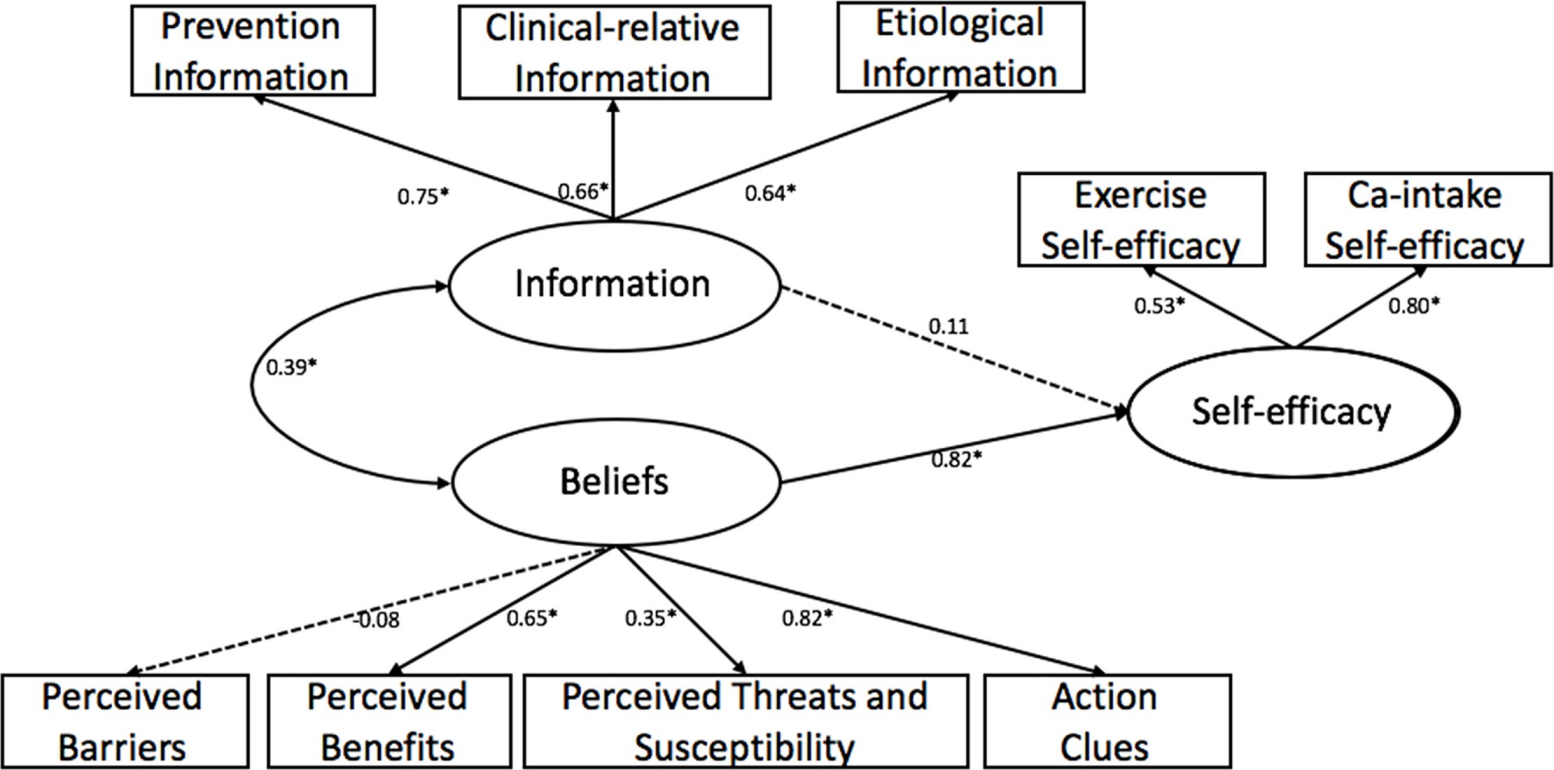
The initial confirmatory pathway, predicting self-efficacy among 421 residuals in Shanghai. Oval represent latent variables; rectangle represent observable variables. Single-headed arrow represent regression path, double-headed arrows represent correlations. Dotted line indicates non-significant path from original model. Regression coefficient are standardized (*p<0.05).

Fig 1 depict the initial pathway model. However, the initial model performed poorly for the participants. The model fit indices were as follows: χ^2^ = 93.361, df = 24, p<0.001, thus the χ^2^/df ratio (χ^2^/df = 3.89) exceeded the acceptable range of 3 or less, the CFI = 0.916, and the RWSEA was not acceptable at 0.083. In a word, the initial model should be modified.

### 3.4 Modified pathway

**Fig 2:**
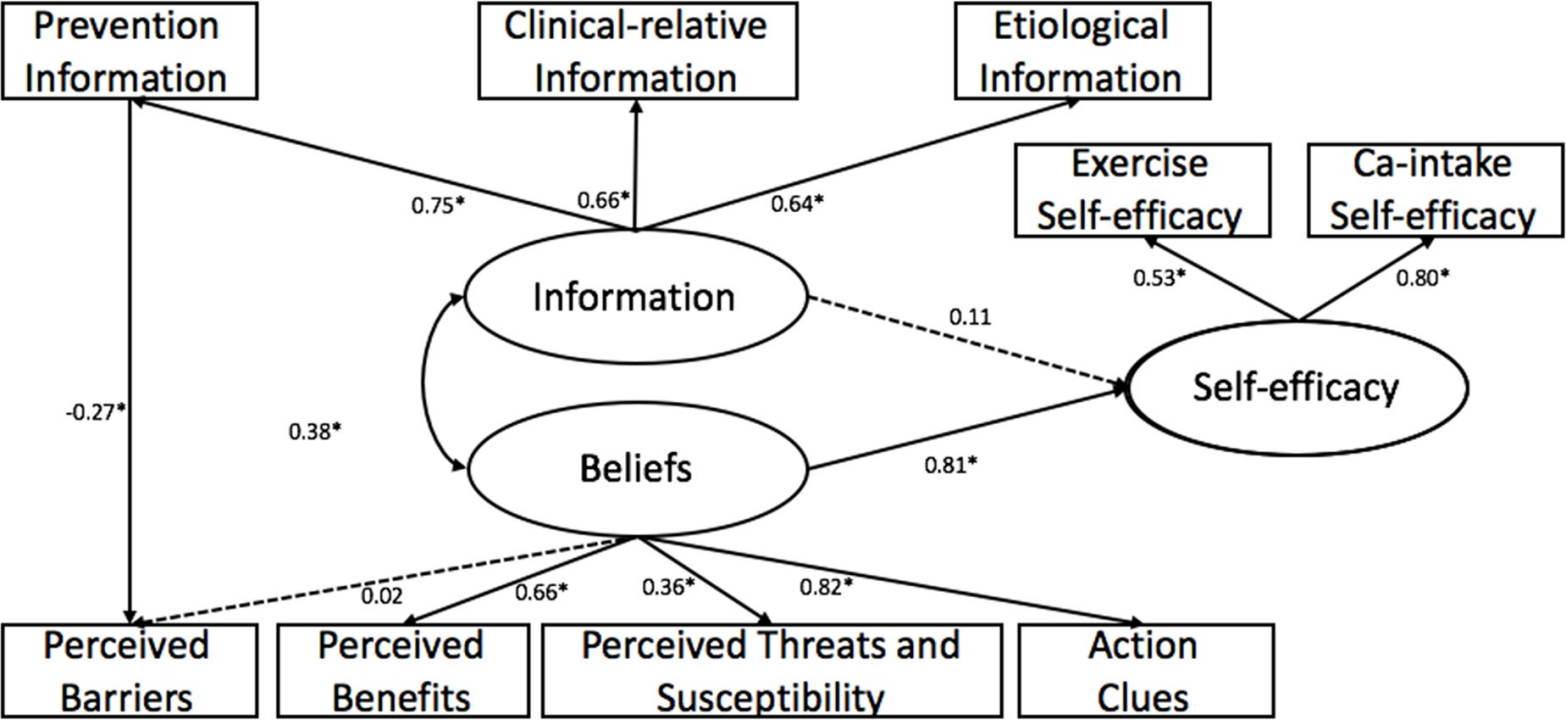
The final confirmatory pathway, predicting self-efficacy among 421 residuals in Shanghai. Oval represent latent variables; rectangle represent observable variables. Single-headed arrow represent regression path, double-headed arrows represent correlations. Dotted line indicates non-significant path from original model. Regression coefficient are standardized (*p<0.05).

We added the supplementary path to the initial pathway model. The final pathway model was shown in Fig 2. After modification, the final pathway model performed well for the participants with the acceptable model fit indices: χ^2^ = 64.123, df = 23, p<0.001, χ^2^/df = 2.788, CFI = 0.95, RMSEA = 0.065.

As the pathway model predicted, beliefs (β = 0.81, p<0.01) had strongly positive effect on self-efficacy, while information was not significant associated with self-efficacy. And information was moderately correlated with beliefs (r = 0.38, p<0.01). Additionally, a new path was added in the final pathway model, namely perceived barriers was significantly negative predicted by prevention information (β = -0.27, p<0.01).

## Discussion

The findings indicated that osteoporosis beliefs were found to strongly affect self-efficacy in performing osteoporosis preventive behaviors among residents over 40 years old, while osteoporosis information was not directly associated with self-efficacy. Information was moderately correlated with beliefs and to indirectly affect self-efficacy through affecting beliefs. In the study, the beliefs were strongly associated with self-efficacy among middle-aged and older community residents, which echoed previous research studies [20].

In this study, gender and employment status were found to significantly affect self-efficacy in the social-demographic part. Previous studies also showed female believed more strongly than men in self-efficacy with respect to osteoporosis screening [34]. At the same time, the participants who were not at work showed better self-efficacy compared with participants who were at work. Perhaps residents with no jobs have more disposable time and are more likely to develop and implement programs to improve their diet, exercise and body care.

In linear regression, information and beliefs showed a statistical significance, which was consistent with previous research results [21]. However, we thought the variables in both studies may exhibit a multicollinearity problem due to the limit of data or the association between information and beliefs, so we decided to deeply study the pathways among information, beliefs and self-efficacy by applying SEM. The pathway model showed that beliefs could strongly affect self-efficacy while information was not directly associated with self-efficacy, which differed from that of previous researches. A research by Shin et al reported that there were significant positive correlations between knowledge and self-efficacy among the adult population with an average score of 11.10/24 on osteoporosis knowledge [35]. Hsieh et al found that knowledge of osteoporosis was positively correlated with self-efficacy for calcium intake and exercise among residents whose mean knowledge of osteoporosis score was 12.07/20, and a mean age of 46.51 years [36]. Perhaps the inconsistency between their studies and this study was caused by their neglect of the effect of beliefs. Or maybe it was because of the limitation of the information’s impact on self-efficacy. When people's osteoporosis-related awareness is at a relatively low level, improving the level of information could have a positive impact on self-efficacy. If people have already had a certain level of osteoporosis awareness, it would be difficult to significantly improve self-efficacy by increasing the information available.

In the pathway model, perceived barriers was significantly negative predicted by prevention information. If people have high level of prevention information, there may be lowered perceived barriers so perceived barriers have limited effect on beliefs. When people don't know much about osteoporosis, they may first think of its benefits rather than barriers. Therefore, if people are willing to adopting suitable behaviors to prevent osteoporosis, improving their beliefs, including perceived benefits, perceived threats, and action clues, is warranted.

Our findings should be interpreted in the light of several limitations. As it was cross-sectional, it is impossible to assess changes in self-efficacy following improvements in information and beliefs. The study used a self-administered questionnaire, and thus there may have some bias in information comprehension. However, our investigators were professionally trained to explain the issues to the residents, and finally could improve their understanding and minimize the information bias. The object of the study was to investigate community residents in Shanghai, but the study did not cover the whole community. There may be discrepancies in socio-demographic or other potential factors between Shanghai and other cities in China. Further unbiased studies and a cohort study in a larger population should be done to confirm the results. There might be other contents associated with self-efficacy not included in our model. Evidence showed that risk information seeking may indirectly process the development of preventive behaviors[37] but this item was not included in our study. Further studies may focus on how the ability of seeking information affect osteoporosis prevention behaviors in community residents. Finally, the study reflects the association and pathway between knowledge and belief and self-efficacy, but could not examine or identify causal relationships because of its design.

Overall, our study is the first, to our knowledge, to analyze the association of information and beliefs with self-efficacy and their pathway among middle-aged and older residents in China. The results suggest that institutions should pay more attention to strengthening osteoporosis-related beliefs.

## Conclusions

Osteoporosis beliefs, especially perceived benefits, perceived threats, and action clues, can strongly affect self-efficacy in performing osteoporosis preventive behaviors, while information was not significant directly associated with self-efficacy. The results highlight the importance of promoting actions to prevent osteoporosis among middle-aged and older residents over 40 years old in China as the lack of information and low level of beliefs and self-efficacy about osteoporosis prevention. It also suggests that future interventions should focus on improving beliefs in ability to take preventive measures as the final path model indicates.
